# Health literacy level in a various nephrology population from Québec: predialysis clinic, in-centre hemodialysis and home dialysis; a transversal monocentric observational study

**DOI:** 10.1186/s12882-021-02464-1

**Published:** 2021-07-09

**Authors:** Annabel Boyer, Yannick Begin, Julie Dupont, Mathieu Rousseau-Gagnon, Nicolas Fernandez, Maryam Demian, David Simonyan, Mohsen Agharazii, Fabrice Mac-Way

**Affiliations:** 1 CHU de Queébec Research Center, L’Hôtel-Dieu de Québec Hospital, Québec, QC Canada; 2 Division of Nephrology, Faculty of Medicine, Université Laval, Québec, QC Canada; 3 Centre Universitaire des Maladies Rénales, CHU de Caen, 14033 Caen Cedex 9, France; 4 Nurse practitioner, CHU de Québec-Université Laval Nursing Department, L’Hôtel-Dieu de Québec Hospital, Québec, QC Canada; 5 Department of Family Medicine and Emergency Medicine, Université de Montréal, Québec, Canada; 6 Department of Psychology, Simon Fraser University, Burnaby, British Columbia Canada; 7 Clinical and Evaluative Research Platform, CHU de Québec-Université Laval Research Center, Québec, Canada

**Keywords:** Health literacy, Hemodialysis, Peritoneal dialysis, Chronic kidney disease

## Abstract

**Background:**

Health literacy refers to the ability of individuals to gain access to, use, and understand health information and services in order to maintain a good health. It is especially important in nephrology due to the complexity of chronic kidney disease (CKD). The present study sought to define health literacy levels in patients followed in predialysis clinic, in-center dialysis (ICHD), peritoneal dialysis (PD) and home hemodialysis (HHD).

**Methods:**

This transversal monocentric observational study analysed 363 patients between October 2016 and April 2017. The Brief Health Literacy Screen (BHLS) and the Health Literacy Questionnaire (HLQ) were used to measure health literacy. Multivariate linear regressions were used to compare the mean scores on the BHLS and HLQ, across the four groups.

**Results:**

Patients on PD had a significantly higher BHLS’score than patients on ICHD (*p* = 0.04). HLQ’s scores differed across the groups: patients on HHD (*p* = 0.01) and PD (*p* = 0.002) were more likely to feel understood by their healthcare providers. Compared to ICHD, patients on HHD were more likely to have sufficient information to manage their health (*p* = 0.02), and patients in the predialysis clinic were more likely to report high abilities for health information appraisal (*p* < 0.001).

**Conclusion:**

In a monocentric study, there is a significant proportion of CKD patients, especially in predialysis clinic and in-centre hemodialysis, with limited health literacy. Patients on home dialysis (HHD and PD) had a higher level of health literacy compared to the other groups.

**Supplementary Information:**

The online version contains supplementary material available at 10.1186/s12882-021-02464-1.

## Background

Health literacy is defined by the World Health Organization (WHO) as “the cognitive and social skills which determine the motivation and ability of individuals to gain access to, understand and use information in ways which promote and maintain good health” [1]. Encompassed within this definition, is that adequate health literacy requires not only the ability to read and understand information, but also the capacity to actively manage one’s own health, locate and appraise health information, navigate the healthcare system and engage with healthcare providers, to maintain one’s own health and the health of one’s family [2]. The complexity of healthcare and health messages led to the development of health literacy questionnaires as tools to assess patients’ health information understanding, in order to improve health promotion [3–5].

Health literacy is especially important in nephrology due to the complexity of the high number of comorbidities, and the need for self-management skills [5, 6]. These include adherence to a restrictive diet as well as a complex medication regimen, and attendance at multiple appointments with healthcare providers. Furthermore, patients with end-stage kidney disease (ESKD) are required to make important decisions about the specific type of kidney replacement therapy (KRT) they would like to undergo (in-centre hemodialysis, peritoneal dialysis or home hemodialysis).

There seems to be a wide variability of health literacy levels across CKD stages and groups of patients on different KRT modality [5, 7, 8]. It is estimated that 18–28% of CKD patients have limited health literacy [5, 6, 9–11], which has been associated with poorer outcomes. Findings are similar in patients on dialysis, with a prevalence ranging from 16 to 32% [5–7, 12–15] and 6–50% [6, 16, 17] for patients on hemodialysis and peritoneal dialysis (PD) respectively. As the nature of home dialysis requires self-management and understanding of their treatment, one could expect a higher health literacy level in home dialysis patients. The wide variation observed could be explained by differences in population or health literacy assessment tools.

The present study sought to define health literacy levels in French-speaking CKD patients on predialysis clinic, in-center hemodialysis (ICHD), PD and home hemodialysis (HHD) at CHU de Québec – Université Laval, using two health literacy measures (Brief Health Literacy Screen and Health Literacy Questionnaire). We used two different questionnaires to consolidate our results. We hypothesized that the health literacy level would be low in our population and that there would be differences across those groups, with a lower health literacy level in patients on ICHD compared to the other groups.

## Methods

### Study design and population

This transversal monocentric observational study was conducted at CHU de Québec – l’Hôtel Dieu de Québec Hospital from October 2016 to April 2017. Eligible participants were adults aged ≥18 years old, who spoke fluent French, capable of providing informed consent, followed in our predialysis clinic (CKD stage 4 and 5) or undergoing dialysis (ICHD, PD, or HHD) for at least 3 months. All eligible participants were approached and offered to participate in the study. They were included after providing an informed consent. Patients with a medical diagnosis of dementia, psychosis or cognitive impairment were excluded from participation. Of note, all patients followed in our predialysis clinic had received an explanation on the different KRT. Therapeutic educational sessions were not available in our centre.

### Data collection

Selected patients received self-administered and voluntary questionnaires. Two questionnaires were used to measure health literacy: the Brief Health Literacy Screen (BHLS) to screen for low health literacy, and the Health Literacy Questionnaire (HLQ) to evaluate multiple health literacy domains. There was no time limit to complete the questionnaires. Questionnaires were administered by hand, either during the ICHD treatment, or in the waiting room before medical visits. They were collected at the end of the ICHD treatment or after the medical visit. Patients did not receive help from staff members to complete the questionnaires Patients’ data (i.e., age, sex, renal diagnosis, diabetes, hypertension, living status, relationship status, education, children, employment status) were collected from patients’ files. This study was approved by the ethics committee of CHU de Québec, Université Laval and was conducted according to the declaration of Helsinki.

### Brief health literacy screen

The BHLS uses the following self-report questions: (1) How confident are you filling out medical forms by yourself? (2) How often do you have someone help you read hospital materials? and (3) How often do you have problems learning about your medical condition because of difficulty understanding written information? Each question can be answered on a Likert-type scale of 1 to 5. Responses are added to calculate a final health literacy score, with low health literacy defined by a score ≤ 9/15 [18]. This validated tool is easy to use, and can be completed in 1 min [4]. It has already been used in patient on ICHD [19] and has been translated in French-Canadian by a bilingual Professor in English, for the purpose of this study (Supplementary data, Figure 1).

### Health literacy questionnaire

The HLQ is a more contemporary health literacy measure developed in Australia in 2012 using a validity driven approach, and has been translated in several languages, including French-Canadian [20], and used in many countries since then [21, 22]. It has been used in kidney transplant recipients [23] and patients on dialysis [24]. The HLQ was validated on a large sample with robust psychometric analysis conducted, including confirmatory structural equation modeling. The strength of the HLQ is that it consists of 44 items forming nine domains of health literacy [25], which assess the skills needed for adequate health literacy encompassing the WHO’s definition. The first five domains are scored on a 4-point Likert-type response scale (Strongly disagree, Disagree, Agree, Strongly agree) and the last four domains are scored on a 5-point response scale which rates the level of difficulty in undertaking a task (from Cannot do to Very easy) (Supplementary data, Table 1). These nine domains generate comprehensive profiles of the health literacy of individuals and groups. This questionnaire has the advantage of constructing a profile of the various health literacy domains which yields specific information for understanding patients’ individual weaknesses and strengths. It is therefore more useful for a better characterization of differences across our four populations (pre-dialysis clinic, ICHD, HHD and PD).

### Statistical analysis

Continuous variables were described by the mean ± standard deviation (SD), median and interquartile range (IQR), and categorical variables were described by frequencies and percentages. The BHLS and HLQ’s were compared by different factors using parametric (Student’s t test, ANOVA) as well as non-parametric tests (Mann-Withney U test, Kruskal-Wallis test) when appropriate, after normality verification. The generalized linear regression models were fitted to compare the mean scores on the BHLS, as well as scores on the nine health literacy domains of the HLQ, across the four groups (predialysis clinic patients, ICHD, PD, and HHD) after residual’s distribution verification. Multiple linear regression analyses were performed to identify association between the BHLS and HLQ scores and the four different groups, adjusting on patient’s characteristics. In case of multiple comparisons, Tukey-Kramer adjustment was applied. Missing data were less than 5% and considered as missing at random. Analyses were performed with SAS v.9.4 (SAS Institute, Cary, NC, USA), with a two-sided significance level set at *p* < 0.05.

## Results

### Patient characteristics

Of the 456 nephrology patients screened from October 2016 to April 2017, 363 (80%) completed the questionnaires. Of these, 152 (42%) were followed in the predialysis clinic, 157 (43%) on ICHD, 38 (10%) on PD and 16 (5%) on HHD. The flow chart is displayed in Supplementary data, Figure 2. Patients’ characteristics are described in Table 1. Compared to PD and HHD patients, those on ICHD and in the predialysis clinic seemed to be older. Hypertension was equally prevalent across all groups ranging from 90 to 95%. Diabetes seemed to be more prevalent in patients on HD and those in the predialysis clinic. In our population, patients on PD were less likely to live alone. Employment status was lower in patients on ICHD (7.6%) and was higher for those on HHD (56.2%).

**Table 1:** Population characteristics

Variables	Predialysis clinic ***n*** = 152	In center hemodialysis ***n*** = 157	Peritoneal dialysis ***n*** = 38	Home hemodialysis ***n*** = 16
Gender (male), n (%)	91 (59.9%)	99 (63.1%)	23 (60.5%)	7 (43.8%)
Age in years, median (IQR)	68 (62–77)	72 (62–78)	59 (48–70)	51.5 (43–57)
Diabetes, n (%)	74 (48.7%)	70 (44.6%)	11 (28.9%)	2 (12.5%)
Hypertension, n (%)	143 (94.1%)	149 (94.9%)	34 (89.5%)	15 (93.8%)
Renal diagnosis, n (%)				
Diabetes	55 (36.2%)	44 (28%)	10 (26.3%)	2 (12.5%)
Hypertension	24 (15.8%)	28 (17.8%)	4 (10.5%)	0 (0%)
Glomerulonephritis	20 (13.1%)	31 (19.8%)	7 (18.4%)	5 (31.3%)
Other	53 (34.9%)	54 (34.4%)	17 (44.8%)	9 (56.2%)
Demographic characteristics				
Live alone, n (%)	47 (30.9%)	49 (31.2%)	4 (10.5%)	5 (31.3%)
Education < 11 years, n (%)	46 (30.3%)	74 (47.1%)	16 (42.1%)	1 (6.3%)
Active employment status, n (%)	36 (23.7%)	12 (7.6%)	18 (47.4%)	9 (56.2%)
Marital status				
Single, n (%)	19 (12.5%)	39 (25.2%)	6 (15.8%)	4 (25%)
Married or civil union, n (%)	92 (60.5%)	80 (51.6%)	29 (76.3%)	10 (62.5%)
Divorced, n (%)	19 (12.5%)	15 (9.7%)	2 (5.3%)	1 (6.3%)
Widowed, n (%)	22 (14.5%)	21 (13.5%)	1 (2.6%)	1 (6.3%)

Values are expressed as median (interquartile range) or number (percentage)

*IQR* interquartile range

We observed a high variability in refusal rates for participation; only one (5%) patient on HHD and four (8%) on the PD refused to participate in the study, whereas 40 (21%) in predialysis clinic and 48 (28%) on ICHD refused to participate. In pre-dialysis, among patients who were excluded from the study, 10 (25%) were not able to read, 5 (12.5%) had cognitive impairment, 5 (12.5%) had psychiatric disease and 2 (5%) had intellectual deficiency (Supplementary data, Figure 2).

### Brief health literacy screen

In the univariate analysis, the BHLS’ score was statistically different across the groups, with a mean score (±SD) of 11.4 (±2.59) for patients in the predialysis clinic; of 10.9 (±3.37) for patients on ICHD; of 12.2 (±2.04) for patients on PD; and of 13.4 (±1.63) for patients on HHD (*p* < 0.001). Patients in the predialysis clinic (14.4%) and those on ICHD (29.3%) were more likely to be classified as having low literacy (BHLS≤9) (*p* < 0.001) (Table 2). In the multivariate analysis adjusted on patients’ characteristics, living, relationship, education and employment status; patients on PD had a significantly higher score than patients on ICHD (coefficient estimate at 1,01; *p* = 0.04). An education until at least 11 years was strongly associated with a higher BHLS’ score (coefficient estimate at 0.21, *p* < 0.001).

**Table 2:** Brief Health Literacy Screen results, and comparison of the scores across the four groups of patients using a univariate generalized linear regression model

Variables	Predialysis clinic ***n*** = 152	In center hemodialysis ***n*** = 150^a^	Peritoneal dialysis ***n*** = 38	Home hemodialysis ***n*** = 16	***P***
**Question 1, mean (±SD):** How confident are you filling out medical forms by yourself?	3.66 (±0.98)	3.46 (±1.31)	3.84 (±0.92)	4.19 (±0.98)	0.116
**Question 2, mean (±SD):** How often do you have someone help you read hospital materials?	3.85 (±1.20)	3.71 (±1.30)	4.21 (±0.74)	4.56 (±0.63)	< 0.001
**Question 3, mean (±SD):** How often do you have problems learning about your medical condition because of difficult understanding written information?	3.91 (±1.03)	3.74 (±1.23)	4.16 (±0.75)	4.69 (±0.48)	0.006
**Total score (max 15), mean (±SD)**	11.41 (±2.59)	10.92 (±3.37)	12.21 (±2.04)	13.44 (±1.63)	0.004
**Low health literacy** **BHLS ≤ 9, n (%)**	22 (14.4%)	44 (29.3%)	4 (10.5%)	0 (0%)	< 0.001

Values are expressed as mean ± standard deviation or number (percentage)

*BHLS* Brief health literacy screen, *SD* standard deviation

^a^Of the 157 participants on IHCD, 7 did not answer the BHLS

### Health literacy questionnaire

HLQ scores according to the patients’ group is displayed in Table 3. In the univariate analyses, the scores of the 1st, 2^d^, 3^d^, 5th, 8th and 9th domains of the HLQ were statistically different across the four groups, patients on HHD scoring higher than the other groups. After adjusting for demographic characteristics, the HLQ scores remained statistically different across the four groups of patients (Table 4). Compared to patients on ICHD, patients on PD (*p* = 0.002) and HHD (*p* = 0.01) were more likely to feel understood and supported by their healthcare providers; with a mean score (±SD) of 3.25 (±0.52) for patients in ICHD, of 3.51 (±0.46) for patients on PD and of 3.69 (±0.38) for patients on HHD. Patients on HHD were more likely to have sufficient information to manage their health (*p* = 0.02). Patients in the predialysis clinic were more likely to report high abilities for health information appraisal (maximum score 4), with a mean score of 3.02 (±0.47) compared to patients on ICHD (3.24 ± 0.43) (*p* < 0.001). Finally, patients in the predialysis clinic were less likely to feel confident in understanding health information well enough to know what to do (maximum score 5), with a mean score (±SD) of 3.90 (±0.66), compared to patients on ICHD (4.07 ± 0.62) (*p* = 0.02). An education until at least 11 years old and being married were strongly associated with a higher score in several health literacy domains (data not shown). Older patients were less likely to easily navigate the healthcare system (*p* = 0.02) and understand health information well enough to know what to do (*p* = 0.01). The same comparisons performed using non-parametric tests provided similar results.

**Table 3:** Health Literacy Questionnaire scores across different patient groups in Nephrology

Parameters	Predialysis clinic ***n*** = 152	In center hemodialysis ***n*** = 157	Peritoneal dialysis ***n*** = 38	Home hemodialysis ***n*** = 16
**HLQ scales, mean (±SD)**	**Range 1 (lowest) to 4 (highest)**			
**Domain 1** Feeling understood and supported by healthcare providers	3.27 (±0.41)	3.26 (±0.52)	3.51 (±0.46)	3.69 (±0.38)
**Domain 2** Having sufficient information to manage my health	3.17 (±0.46)	3.20 (±0.47)	3.26 (±0.49)	3.50 (±0.52)
**Domain 3** Actively managing my health	3.09 (±0.43)	3.16 (±0.43)	3.15 (±0.43)	3.38 (±0.27)
**Domain 4** Social support for health	3.19 (±0.43)	3.20 (±0.50)	3.34 (±0.37)	3.43 (±0.43)
**Domain 5** Appraisal of health information	3.24 (±0.43)	3.02 (±0.47)	2.96 (±0.46)	3.11 (±0.46)
	**Range 1 (lowest) to 5 (highest)**			
**Domain 6** Ability to actively engage with healthcare providers	4.00 (±0.58)	3.98 (±0.62)	4.09 (±0.57)	4.28 (±0.61)
**Domain 7** Navigating the healthcare system	3.90 (±0.62)	3.90 (±0.62)	3.92 (±0.57)	4.05 (±0.53)
**Domain 8** Ability to find good health information	3.91 (±0.68)	3.87 (±0.60)	3.79 (±0.53)	4.19 (±0.46)
**Domain 9** Understand health information well enough to know what to do	3.90 (±0.66)	4.07 (±0.62)	4.19 (±0.58)	4.46 (±0.45)

Values are expressed as mean ± standard deviation

*HLQ* health Literacy Questionnaire, *SD* standard deviation

**Table 4:** Results of multivariate linear regressions showing the association between the scores on the nine health literacy domains of the HLQ of the pre dialysis, PD and HHD patients compared to ICHD patients

Parameters	Predialysis clinic***n*** = 152		Peritoneal dialysis***n*** = 38		Home hemodialysis***n*** = 16	
	Coefficient estimate	*P* value	Coefficient estimate	*P* value	Coefficient estimate	*P* value
Domain 1	0.03	0.55	0.28	0.002	0.35	0.01
Domain 2	0.01	0.82	0.11	0.22	0.33	0.02
Domain 3	−0.07	0.20	0.01	0.87	0.12	0.30
Domain 4	−0.02	0.71	0.14	0.12	0.15	0.23
Domain 5	0.20	< 0.001	−0.04	0.62	0.07	0.56
Domain 6	0.02	0.75	0.14	0.20	0.19	0.25
Domain 7	0.05	0.53	0.05	0.65	0.07	0.70
Domain 8	0.06	0.46	−0.06	0.60	0.14	0.41
Domain 9	−0.17	0.02	0.10	0.39	0.18	0.30

Multivariate linear regressions adjusted on age, gender, renal disease, diabetes, hypertension, demographic characteristics, education status, active employment status, marital status

*HLQ* health literacy questionnaire, *PD* peritoneal dialysis, *HHD* home hemodialysis, *ICHD* in centre hemodialysis

## Discussion

This study is the first to characterize and compare health literacy levels in a large group of pre-dialysis and in three groups of dialysis patients using two health literacy measures. The findings of the current study reveal differences in health literacy levels between patients in pre-dialysis, ICHD, PD and HHD. Findings on the two health literacy measures paralleled one another, with patients on home KRT (PD and HHD) scoring higher on the BHLS and generally achieving higher scores across several domains of the HLQ.

The lower prevalence of limited health literacy levels in home dialysis patients (PD 10.5% and HHD 0%) compared to patients in the pre-dialysis clinic (14.4%) and on ICHD (29.3%), confirms our initial hypothesis. Our results are also consistent with previous studies, which reported a prevalence of limited health literacy ranging from 16 to 32% for patients on ICHD [5–7, 12–14], and from 6 to 50% for patients on PD [6, 16, 17]. On the contrary, our predialysis population seems to have a better health literacy level compared to other cohort where a prevalence of 18 to 28% was reported [5, 6, 9–11]. To our knowledge, health literacy levels in patients on HHD has not been described before our study.

These results were expected since the nature of home dialysis requires that these patients rely more on their self-management and care skills, proactivity, and have a thorough understanding of the therapy. Even though the results were not statistically significant, patients on HHD tended to score higher on the BHLS than ICHD patients, however the small size of the group could explain why our results did not reach statistical significance. Moreover, the patients on home dialysis (PD and HHD), scoring higher on the BHLS, were younger, had a higher rate of education and of active employment status compared to the other groups. In previous studies, a lower educational attainment was associated with limited health literacy levels in ESKD and ICHD patients [5, 7, 10, 13, 19]. One may also argue that the higher health literacy scores observed in home dialysis patients might favour the choice of KRT towards home dialysis. This hypothesis would need further investigations to be confirmed.

Differences in the several domains of the HLQ have been found between the four groups. For example, patients on home KRT were more likely to feel understood and supported by their healthcare providers, which could be explained by a closer relationship between patients and the home dialysis healthcare professionals. Indeed, most patients on home KRT establish a trusting relationship with their healthcare providers, and have access to a 24 h/7 days phone health care support. On the other hand, patients from the pre-dialysis clinic may see different nephrologists at each appointment and patients on ICHD may be followed by a different nephrologist each week.

Patients in the predialysis unit were more likely to report high abilities for health information appraisal. This could be explained by the fact that they have less to learn since explanations are provided on a regular basis and that they are provided with a book containing information on their CKD condition [26]. There was no difference in the ability to find good health information among groups. Finally, patients in the predialysis clinic were less likely to feel confident in understanding health information well enough to know what to do compared to patients on ICHD. This could be explained by the smaller frequency of contact with healthcare professionals for patients in the predialysis clinic, compared to patients on ICHD who are followed up to three times a week by the medical team.

HHD is more complex than PD, which explains why this group scored higher on many domains of the HLQ. Peritoneal dialysis is a relatively easy procedure, which may explain why as many as 42% of PD patients have low education, 10.5% were identified as having low health literacy on the BHLS, and patients could be as old as 83 years old. Although health literacy levels in the ICHD group are lower, many patients do have a high literacy level, suggesting that patients may choose ICHD for reasons unrelated to the health literacy levels (lack of space for HHD, feeling of safety for ICHD, not wanting to bring CKD at home).

Recent literature supports that health literacy levels in CKD patients may be associated with clinical outcomes. It has been reported that participants with limited health literacy had lower estimated glomerular filtration rate [8] and higher self-reported cardiovascular diseases [11]. In the ICHD population, limited health literacy was independently associated with an increased incidence of emergency department visits and dialysis related hospitalizations [14], increased risk of death [7], a lower risk of being referred for transplant evaluation [12], and worse blood pressure control than those with adequate health literacy [15]. In PD patients, limited health literacy was not associated with an increased risk of infectious complications or hospitalization [17]. The authors suggested that these results were related to the rigorous training that PD patients had to undergo, and that limited health literacy should not prevent patients from being considered eligible for PD [6, 17].

Younger age and being married were associated with a higher score in several health literacy domains of the HLQ. Similar findings have been described previously [7, 9, 15, 19].

Limitations of the present study include its descriptive, observational, monocentric nature, as well as the use of self-reported measures of health literacy. The high variability in refusal rates for participation may have underestimated the prevalence of low health literacy. This is especially true in the ICHD group where 25% of the patients refused to complete the HLQ. Finally, as the BSHL was only validated in English, it has been translated in French-Canadian by a bilingual Professor. However, this French version has not been validated. For future research, it would be of interest to validate this instrument in order to support the external validity of our study.

Our study has several strengths. The high participation rate of patients on home dialysis (91%) enables us to provide a first reliable assessment of health literacy in this population, even though this estimation could be biased by the fact that HHD patients might be more educated and less reluctant to fill in questionnaires. We are therefore confident that our results will be useful for conducting future studies on health literacy in home dialysis patients. We used a validated health literacy tool (HLQ) with unique assessment of nine domains of health literacy. In contrast to older measures (REALM [27], TOFHLA [28]), its assessment goes beyond the domains of reading and writing ability, and matches more closely the definition of health literacy adopted by the WHO. Also, the French version of the HLQ was found to have very good internal consistency across the nine domains of health literacy [22].

In conclusion, there is a significant proportion of CKD patients, especially in predialysis clinic and in-centre hemodialysis, with limited health literacy. Development and update of education tools and methods in Nephrology will need to take into account the limited health literacy that is prevalent in our patients. A systematic health literacy level screening could be implemented in nephrology centres. As mentioned by the Assistant Secretary of Health, Howard Ko “The responsibility is ours as health professionals to communicate in plain language. Without clear communication, we cannot expect people to adopt the healthy behaviors and recommendations that we champion.” [29].

## Supplementary Information

**Additional file 1.**

## Data Availability

All data generated or analysed during this study are available from the corresponding author on reasonable request.

## Abbreviations

BHLS: Brief Health Literacy Screen
CKD: Chronic kidney disease
ESKD: End stage kidney disease
HHD: Home hemodialysis
HLQ: Health Literacy Questionnaire
ICHD: In-center hemodialysis
IQR: Interquartile range
PD: Peritoneal dialysis
KRT: Kidney replacement therapy
SD: Standard deviation
WHO: World Health Organization
