# Supplementary material for: Health literacy level in a various nephrology population from Québec: predialysis clinic, in-centre hemodialysis and home dialysis; a transversal monocentric observational study
Source: BMC Nephrol. 2021 Jul 9;22:259. doi: 10.1186/s12882-021-02464-1 (PMC8272301; doi:10.1186/s12882-021-02464-1)
Supplement: Supplementary file 1 — Additional file 1. [file 12882_2021_2464_MOESM1_ESM.doc]

**Supplementary Data**

**Title:**Health literacy level in a various nephrology population from Québec: predialysis clinic, in-centre hemodialysis and home dialysis; a transversal monocentric observational study.

**Authors**: Annabel BOYER MD^1,2,3*^ and Yannick BEGIN MD^1,2*^, Julie DUPONT NP^4^, Mathieu ROUSSEAU-GAGNON MD^1,2^, Nicolas FERNANDEZ PhD^5^, Maryam DEMIAN PhD^6^, David SIMONYAN MSc^7^, Mohsen AGHARAZII MD^1,2^, Fabrice MAC-WAY MD^1,2^

^*^Dr Annabel Boyer and Yannick Bégin have contributed equally to this work.

^1^CHU de Québec Research Center, L’Hôtel-Dieu de Québec Hospital, Québec, QC, Canada 
^2^Division of Nephrology, Faculty of Medicine, Université Laval, Québec, QC, Canada

^3^Centre Universitaire des Maladies Rénales, CHU de Caen. 14033 Caen Cedex 9, France

^4^Nurse practitioner, CHU de Québec-Université Laval Nursing Department, L'Hôtel-Dieu de Québec Hospital, QC, Canada CA

^5^Department of Family Medicine and Emergency Medicine, Université de Montréal, Québec, Canada

^6^Department of Psychology, Simon Fraser University, Burnaby, British Columbia, Canada

^7^Clinical and Evaluative Research Platform, CHU de Québec-Université Laval Research Center, Québec, Canada

Correspondence:

Annabel Boyer

L'Hôtel-Dieu de Québec Hospital

11 Côte-du-Palais

Quebec, Canada, G1R2J6

E-mail: [annabel.boyer@wanadoo.fr](mailto:annabel.boyer@wanadoo.fr)

Tel: 418 691-5464

Fax: 418 691-5562

**Figure S1:** BHLS Questionnary

Questionnaire clinique

Évaluation de votre situation

Nom : ___________________________________

Âge : ___________________________________ ans

Sexe : □ Homme □ Femme

Habitez-vous seul : □ oui □ non

Langue maternelle □ Français □ Anglais □ autre

Race □ Blanc □ Autochtone

□ Autre : ­___________________________

□ Célibataire □ union civil ou marié

□ divorcé □ veuf/veuve

Combien d’enfant avez-vous? _________________

Nombre d’années d’éducation : _______________ ans

□ Employé : __________________________

□ Étudiant : ___________________________

□ Recherche d’emploi □ retraité

□ Autre : _______________________

1) À quel point être vous confiant de pouvoir remplir des formulaires médicaux par vous-même?

| Pas confiant  □ | Un peu confiant  □ | Confiant  □ | Très confiant  □ | Extrêmement confiant  □ |
| --- | --- | --- | --- | --- |

2) Avez-vous souvent besoin de l’aide de quelqu’un pour lire du matériel médical ?

| Tout le temps  □ | La plupart du temps  □ | Parfois  □ | À quelques occasions  □ | À aucune occasion  □ |
| --- | --- | --- | --- | --- |

3) Avez-vous souvent de la difficulté à en apprendre plus sur votre condition médicale parce que les informations écrites sont difficiles à comprendre ?

| Tout le temps  □ | La plupart du temps  □ | Parfois  □ | À quelques occasions  □ | À aucune occasion  □ |
| --- | --- | --- | --- | --- |

**Figure S2~~1~~:** Flow chart

Screened population

Predialysis

n= 192

ICHD

n= 205

PD

n= 42

HHD

n= 17

**Predialysis**

**n= 152**

**ICHD**

**n= 157**

**PD**

**n= 38**

**HHD**

**n= 16**

**Excluded = 40**

Refusal: 10

Illiterate: 10

Language: 5

Vision: 3

Cognitive: 12

**Excluded = 48**

Many unknown,

Illiterate,

Fatigue,

Vision

**Excluded = 4**

Illiterate: 1

Language: 2

Unknown: 1

**Excluded = 1**

Unknown: 1

**Table S1:** Health Literacy Questionnaire Scales

| **Scales** | **Number of items** |
| --- | --- |
| **Domain 1** Feeling understood and supported by healthcare providers | 4 |
| **Domain 2** Having sufficient information to manage my health | 4 |
| **Domain 3** Actively managing my health | 5 |
| **Domain 4** Social support for health | 5 |
| **Domain 5** Appraisal of health information | 5 |
| **Domain 6** Ability to actively engage with healthcare providers | 5 |
| **Domain 7** Navigating the healthcare system | 5 |
| **Domain 8** Ability to find good health information | 5 |
| **Domain 9** Understand health information well enough to know what to do | 5 |

Scales 1-5 are rated on a 4-point Likert-type scale (1: strongly disagree to 4: strongly agree) and Scales 6-9 on a 5-point Likert-type scale (1: cannot do to 5: very easy)
